# Threat Agnostic Approach to Epidemic Management Using Continuous Remote Patient Monitoring

**DOI:** 10.1089/hs.2023.0061

**Published:** 2024-04-16

**Authors:** Arik Eisenkraft

**Affiliations:** Arik Eisenkraft, MD, MHA, is a Senior Researcher, The Institute for Research in Military Medicine, The Department of Military Medicine, The Hebrew University Faculty of Medicine and the IDF Medical Corps, Jerusalem, Israel.

**Keywords:** Remote patient monitoring, Threat agnostic approach, Biothreat agents, Bioterror, National security

## Introduction

Various technologies have been developed in response to the increasing prevalence of biological threats, some of which are threat agnostic, meaning they can protect against and counter multiple types of bioagents.^1-5^ Novel technologies for the full and accurate characterization of host damage and immunological status are becoming increasingly robust, due to the introduction of new techniques and tools, such as advanced imaging and analytical methods. These allow for a more precise and detailed analysis of the specimens being studied and provide a greater understanding of the different components of the immune system and how they interact with the environment. Furthermore, the use of high-throughput sequencing technology has enabled researchers to identify novel biomarkers of tissue damage and immune system dysfunction.^4,5^

An effective threat agnostic system for epidemic management must be able to detect and respond to a wide variety of threats in an automated manner. Such a system requires access to a wide variety of threat data sources and the ability to process the data effectively and efficiently, identify any patterns, and quickly adapt to new threats. Furthermore, it should effectively prioritize threats and respond to them promptly, which is more relevant for deliberate events where multiple simultaneous threat agents may be used. This would allow health systems and other national security agencies to improve situational awareness and better manage available resources for both immediate and long-term responses. Lastly, the system must be able to scale quickly to meet the demands of a rapidly changing threat landscape. Developing and implementing such a threat agnostic approach to epidemic management requires overcoming several technical challenges and gaps. In the next decade, improved technologies will be key in enabling threat agnostic approaches to counter biological threats and strengthen all aspects of epidemic management. For example, technologies that can support the detection, monitoring, identification, and prevention of biological threats using artificial intelligence (AI)-based machine learning and predictive analytics will be essential, as well as capabilities that enable secured storage of data and communication between multiple parties. These improvements could dramatically shorten the time to achieve full epidemiological investigation. Finally, the use of biometric and genetic data to create a comprehensive database will also be obligatory. Ultimately, these technologies will enable a better understanding of the risks posed by biological threats and improved capability to develop more effective countermeasures in a shorter timeframe.^6,7^

## Continuous Remote Patient Monitoring and Host-Based Diagnostics

Recent technological advances in the field of medical-grade wearable devices are transforming the way patients are monitored in everyday life, whether in hospital settings, outpatient clinics, hospital-at-home programs, older adult home care, or at home.^8-11^ As such advanced remote patient monitoring platforms are used more abundantly to monitor patients in both acute and chronic care settings, more people are monitored for longer periods, allowing multiple parameters to be continuously collected and analyzed. This results in new insights, shedding light on physiological trajectories and dynamic changes among various populations with multiple medical conditions. However, this massive amount of data presents some challenges. For wearable technology to be diagnostic, it must sift through all of the data to identify signals amidst the noise. Standard statistical methods used to analyze these vast amounts of physiological data will be insufficient. The next leap forward is using advanced analytical methods, combining continuous monitoring of multiple hemodynamic parameters with AI-based machine learning tools. Such machine learning algorithms could establish what is normal for each individual, facilitating the detection of deviations from one's baseline. Rather than defining “normal” for a population, machine learning on continuously collected data enables us to define “normal” for that user. This is highly important when trying to improve the care of patients with noncommunicable diseases such as hypertension, heart failure, chronic obstructive pulmonary disease, oncology, and others. It becomes even more crucial when aiming to prevent and control infection transmission at an early stage. Such capabilities could shift the pendulum from a reactive approach, in which response efforts start after an infectious agent is already showing its effects in the population, to an approach focused mainly on preventive measures, instituted based on presymptomatic physiological patterns.

In previous research efforts, we demonstrated that continuous monitoring of advanced hemodynamic parameters combined with advanced AI-based machine learning tools can help detect early presymptomatic changes during the early stages of influenza, which could enhance future biosurveillance efforts.^12^ Among the potential benefits of this approach are the timely identification of outbreaks, increased situational awareness, and improved utilization of isolation measures and medical treatments. Overall, this technique has shown the potential to better manage influenza outbreaks and reduce morbidity and mortality associated with the disease. It is expected that more people will use such seamless, wearable, noninvasive monitoring devices during their routine life, providing valuable information on their physiological trajectories and helping clinicians with medical decisions related to their routine health. Huge amounts of data are already being transferred through cloud-based serverless solutions that provide massive capacity for collecting, storing, and assessing the data. Thus, the use of such routine monitoring tools by vast numbers of people could also enable the early detection of physiological changes when a biological threat agent starts spreading, especially on a large scale. Moreover, assuming these patterns will be unique to each threat agent, such tools could allow for the early detection of specific threat agents, not only influenza, and could provide more than a general alert of physiological changes. Early detection of a pathogen (eg, 2 days) should be sufficient to enable the immediate implementation of emergency measures to reduce disease spread and the deployment of rapid response teams, as well as help decisionmakers to move quickly toward developing and producing relevant medical countermeasures. Once a breakout is suspected, more devices could be deployed and distributed to the population to increase understanding about the spread of the disease. To achieve such a system, logistical chains should be highly trained and capable. These capabilities are complex, labor-intense, and should preferably be combined with cutting-edge computational capabilities, allowing full control and situational awareness of the various logistical aspects required in these scenarios. This would ensure that facilities are well stocked with devices that can be moved quickly, anywhere and anytime. Importantly, repeated training is required in order to assure the preparedness plan is practical and that all objectives can be accomplished.

In a study with COVID-19 patients, we showed that monitoring using wearable wireless sensors far exceeded the capabilities of standard monitoring techniques (eg, finger-tip pulse oximeters, manual cuff-based blood pressure devices, counting breaths by healthcare providers) by deciphering early cardio-pulmonary changes resulting from the infection.^13^ In a study focused on the COVID-19 vaccine, we also showed the capability of wireless sensors in tracking physiological changes resulting from vaccines, including the Pfizer BioNTech BNT162b2 mRNA COVID-19 vaccine used during the pandemic.^14^ This also opens up the possibility of creating a loop that integrates wireless sensor monitoring with automatic alert generation and transmission to caregivers. Such a system could significantly enhance the effectiveness of monitoring and enable timely interventions, ultimately leading to better health outcomes for patients. To test this specific possibility, the platform we used integrated continuous remote patient monitoring with AI-based machine learning tools to collect and analyze the multiple physiological parameters automatically collected by such medical-grade monitoring systems. The system automatically calculated and provided several scores of physiological importance, including an early warning score,^15,16^ shock index, compensatory reserve index, and more. By applying AI-based machine learning tools to the collected data, we showed how it is possible to predict and warn of patients' risk to deteriorate several days before the medical teams were aware of it, as well as predict response to therapy in complex ambulatory patients.^15,16,17^ This was shown not only in cardiology patients, but also in patients with chronic obstructive pulmonary disease, pneumonia, and sepsis.^15,16^

We already see that such a monitoring platform can serve as an early detection tool of host response, and it could also be considered an agnostic host-based diagnostic tool to complement pathogen-based diagnostics. When using such physiological monitoring technologies, the biomarkers are not molecular in nature, such as proteins, metabolites, or RNA transcripts; instead, the physiological changes in multiple cardiopulmonary parameters reflect the host response and are measured noninvasively by analyzing high-density data and detecting an individual's deviations from baseline. Beyond presymptomatic detection and continuous monitoring, this platform can aid with ongoing clinical assessment and decisionmaking by providing insights into a patient's clinical status and disease severity, as well as providing trajectories of deterioration to severe outcomes (eg, sepsis).

## Conclusion

The use of continuous remote patient monitoring can serve as an agnostic host-based diagnostic tool to complement pathogen-based diagnostics. It not only has the potential to map an individual patient's status, but it could also help with diagnosis and address existing gaps in the ability to respond to unidentified threats. Moreover, by using remote patient monitoring platforms, such insights can be achieved wherever the patients are located, whether at home or in the hospital setting, further helping with situational awareness of national health systems in cases of biological outbreaks. This will not only help improve clinical care, but it will also fortify national security efforts when managing biological threat agents.
